# Endocardiac Injection of Strophanthin

**Published:** 1916-07

**Authors:** 


**Endocardiac Injection of Strophanthin.** Ruediger, Muench. Med. Woch., Jan. 25, reports the case of an elderly woman with cyanosis, oedema and double hydrothorax. The patient was apparently moribund but was revived and made a relative recovery. He has practiced this method several times since 1909 and advises injection into the base of the right ventricle.

